# Panx1 regulates neural stem and progenitor cell behaviours associated with cytoskeletal dynamics and interacts with multiple cytoskeletal elements

**DOI:** 10.1186/1478-811X-11-62

**Published:** 2013-08-21

**Authors:** Leigh E Wicki-Stordeur, Leigh Anne Swayne

**Affiliations:** 1 Division of Medical Sciences, Island Medical Program, University of Victoria, Victoria, British Columbia, Canada; 2 Department of Biology, University of Victoria, Victoria, British Columbia, Canada; 3 Department of Biochemistry and Microbiology, University of Victoria, Victoria, British Columbia, Canada; 4 Department of Cellular and Physiological Sciences, University of British Columbia, Vancouver, British Columbia, Canada

## Abstract

**Background:**

Pannexins (Panxs) are relatively newly discovered large-pore ion and metabolite permeable channels. Although no proteomics-based interactome has yet been published, Panx1 has been demonstrated to interact with actin in an ectopic expression system. This interaction affects both Panx1 plasma membrane stability as well as cytoskeletal remodelling. The current study builds on our recent discovery of Panx1 expression in ventricular zone (VZ) neural stem and progenitor cells (NSC/NPCs), and on the demonstrated interaction of Panx1 with the cytoskeleton.

**Findings:**

Here we demonstrate that Panx1 also plays roles in two additional cell behaviours associated with neurogenesis, including cell migration and neurite extension. Furthermore, we confirm an endogenous interaction between actin and Panx1, and identify a new interaction with actin-related protein 3, an actin cytoskeleton-modulating protein.

**Conclusions:**

This study further establishes the importance of Panx1 in the cell biology of NSC/NPCs and strengthens and expands our knowledge of Panx1 interactions with the cytoskeleton.

## Findings

Panxs are four-pass transmembrane proteins that oligomerize to form large-pore mechanosensitive channels permeable to ions and metabolites of up to 1 kDa in size, such as adenosine triphosphate (ATP) [1,2]. We recently detected Panx1 expression in the Neuro-2a (N2a) cell line as well as in post-natal ventricular zone (VZ) neural stem and progenitor cells (NSC/NPCs), where it positively regulates cell proliferation in part through release of ATP that results in activation of purinergic receptors. This built on earlier work demonstrating an important role of constitutively released episodic bursts of ATP in the proliferation of VZ NSC/NPCs, which in turn activates metabotropic purinergic receptors in an autocrine and paracrine manner [3-5]. Perhaps not surprisingly, as a mechanosensitive channel, recent work has shown that Panx1 is actually physically linked to the cytoskeleton. In an ectopic expression system, Panx1 was reported to physically interact directly with actin [6]. A recent study in glioma cells further supported a role for Panx1 in the dynamic regulation of actin cytoskeleton remodeling [7]. Here we extend on our previous discovery of Panx1 expression in VZ NSC/NPCs by further defining the cell-type demographics of Panx1 over the course of VZ neurogenesis, by demonstrating that Panx1 plays a role in additional cell behaviours associated with neurogenesis, including cell migration and neurite outgrowth, and by uncovering additional interactions with cytoskeletal elements, further establishing the relationship of Panx1 with the cytoskeleton.

**Figure 1:**
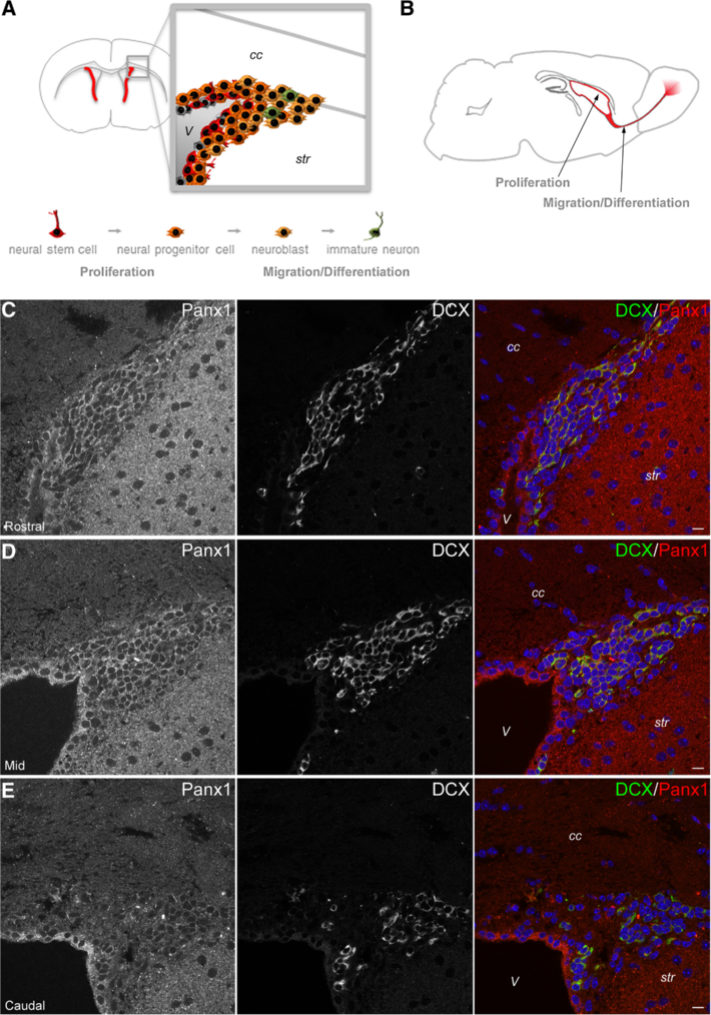
**Panx1 is differentially expressed in VZ migrating neuroblasts. (A)** Cartoon representation of a caudal section, with the ventricular zone magnified to show cell distribution. The progression from NSC/NPC to immature neuron is depicted below, with associated cell behaviours outlined. **(B)** Cartoon representation of a sagittal section with the rostral migratory stream from ventricular zone to olfactory bulb outlined, and associated cell behaviours depicted below. Confocal images of cryosectioned P60 mouse dorso-lateral VZ immunostained for Panx1 and Dcx. Panx1 was more strongly co-expressed in migratory Dcx+ cells in the **(C)** rostral and **(D)** mid VZ compared to **(E)** caudal sections. *V*: ventricle, *cc*: corpus callosum, *str*: striatum. Hoechst 33342 was used as a nuclear counterstain. All scalebars are 10 μm.

**Figure 2:**
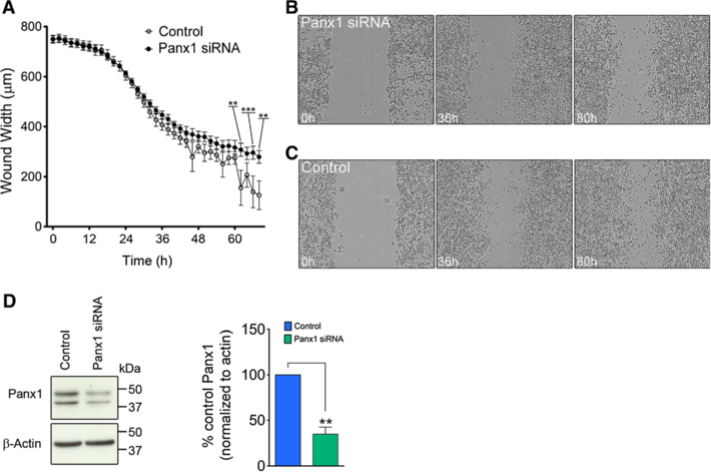
**Panx1 influences cell migration. (A)** Panx1 siRNA knockdown in the N2a cell line caused a reduction in cell migration in a scratch wound closure assay. Wounds were monitored in real time using an Incucyte (Essen Biosciences, Ann Arbour, Michigan, USA). Representative shots of scratch wound closure for **(B)** control and **(C)** Panx1 knockdown N2a cells. **(D)** Western blot of N2a lysates 48 hours post-transfection shows successful Panx1 knockdown (left). The expected Panx1 band is present at ~50 kDa, as well as a lower band that likely corresponds to a lower glycosylation species of Panx1 as it is also specifically knocked down. The percent knockdown at 48 hours was ~60% of control Panx1 levels (right).

**Figure 3:**
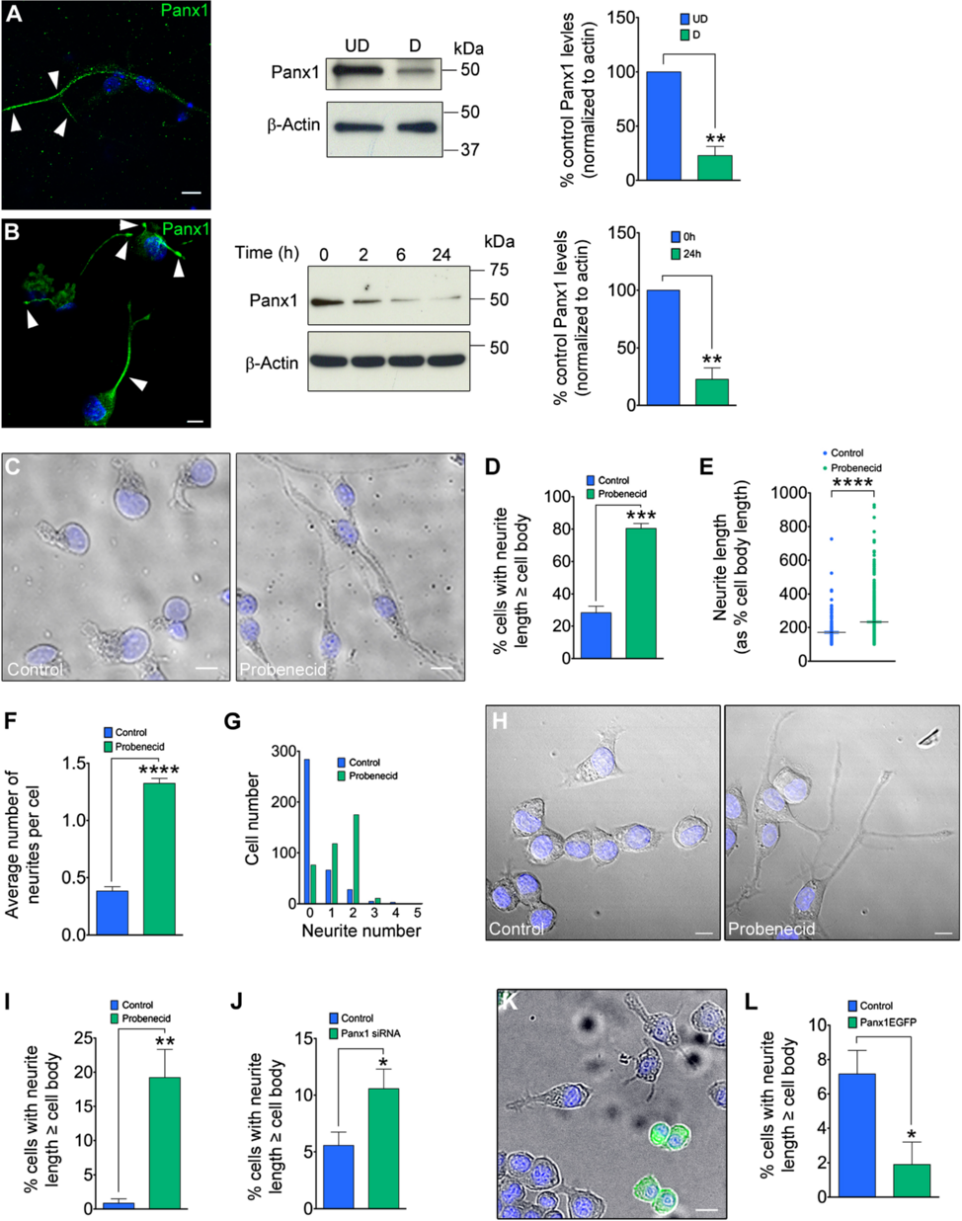
**Panx1 levels decrease across neuronal differentiation and are important for neuritogenesis. (A)** Confocal image of VZ-derived cells under neuronal driving conditions immunolabelled for endogenous Panx1 (left). Arrowheads indicate Panx1 in the neurite. VZ neurospheres were replated and maintained in proliferative conditions (UD; un-differentiated) or neuronal driving conditions (D; differentiated) for 5 days. Panx1 expression assessed by Western blotting (middle) was significantly lower in differentiated compared to undifferentiated neurospheres (right). **(B)** N2a cells were differentiated for 24 hours in low-serum media with 10 μM retinoic acid. Samples were collected at 0, 2, 6, and 24 hours. (Left) Confocal image of endogenous Panx1 staining at 24 hours of differentiation. Arrowheads indicate Panx1 in neurites. Western blotting (middle) revealed significantly reduced Panx1 expression in 24 hour differentiated samples compared to 0 hour controls (right). **(C)** Representative images of VZ NSC/NPCs (dissociated neurospheres) treated with 1 mM probenecid or vehicle control for 48 hours. A process with length greater than or equal to the corresponding cell body length was considered a neurite. **(D)** The percent of VZ NSC/NPCs possessing a neurite increased with probenecid treatment. **(E)** The length of VZ NSC/NPC neurites increased with probenecid treatment compared to control. **(F)** Probenecid treatment significantly increased the average number of neurites per cell in VZ NSC/NPCs compared to control, and **(G)** dramatically altered the neurite number distribution. **(H)** Representative images of N2a cells treated for 36 hours with 1 mM probenecid or vehicle control. **(I)** Probenecid treatment increased the percent of cells possessing a neurite. **(J)** Panx1 siRNA knockdown increased the proportion of cells possessing a neurite. **(K)** Representative image of N2a cells transfected with Panx1EGFP 24 hours after induction of differentiation. **(L)** Significantly fewer N2a cells overexpressing Panx1EGFP possessed one or more neurites compared to untransfected same-plate controls. Hoechst 33342 was used as a nuclear counterstain. All scalebars are 10 μm.

In our previous study [8] we observed marked Panx1 expression in Nestin-positive/glial fibrillary acidic protein (Gfap)-positive and Nestin-positive/Gfap-negative NSC/NPCs, but detected little to no Panx1 expression in doublecortin (Dcx) positive neuroblasts in cultures of differentiating VZ neurospheres, and in Dcx-positive neuroblasts migrating from the dorsolateral corner of the lateral ventricle in coronal sections from immature mice (postnatal day 15; P15). To extend on these findings we investigated the expression of Panx1 in Dcx-positive cells in the adult mouse brain (P60). For a complete description of the methods used in this report, please see Additional file 1. Interestingly, we observed robust Panx1 expression in Dcx-positive cells in rostral coronal sections through the lateral ventricles, but relatively minimal Panx1 in Dcx-positive cells in more caudal coronal sections through the lateral ventricles (Figure 1C-E). The high level of Panx1 expression in rostral Dcx-positive migrating migratory neuroblasts suggested that Panx1 might play a role in modulating the process of cell migration from the VZ. To directly investigate the involvement of Panx1 in cell migration, we employed a scratch wound closure assay [9] monitored in real time (Figure 2A-C), in parallel sets of Panx1 siRNA and control siRNA treated cells. Over time, cell migration into the scratch wound leads to a decrease in width (wound closure), and thus differences in the rate of wound closure can be attributed to differences in cell migration [9]. With a knockdown in Panx1 expression of approximately 60% in Panx1 siRNA-treated cells compared with control siRNA-treated cells (Figure 2D,E), we observed a significant impairment in wound closure. In the corresponding Western blot, the expected Panx1 band is present at ~50 kDa corresponding to the full length, fully glycosylated species [10,11]. The observed lower band was not always present on Western blots of N2a lysates (For example see Figure 3); however when present, it was specifically knocked down by the siRNA. This suggests that it is indeed a form of Panx1 and likely represents one of the lower, less glycosylated species. While we could not track Panx1 knockdown to specific cells, the levels of knockdown we obtained in the overall population provided a significant reduction in cell migration. Together these data suggest that Panx1 plays a role in regulating cell migration.

Dcx-positive neuroblasts overall appeared to express less Panx1 than NSC/NPCs immediately proximal to the ventricle, suggesting that Panx1 expression decreases with differentiation. This was confirmed *in vitro* in retinoic acid and low serum differentiated N2a cells and differentiating VZ NSC/NPC cultures (Figure 3A,B). Interestingly, immunostaining of endogenous Panx1 in neuronally differentiated VZ cells and N2a cells revealed strong Panx1 expression within the developing neurites (Figure 3A,B). Since differentiation of N2a cells and VZ NSC/NPCs *in vitro* is associated with such marked neurite outgrowth, we examined whether blocking or knocking down Panx1 can, on its own, induce neurite extension in the absence of additional differentiation stimuli. Indeed, blocking Panx1 with probenecid [12-14] induced marked neurite extension in both N2a cells and VZ NSC/NPCs (Figure 3C-I). Similarly, Panx1 siRNA knockdown in N2a cells caused increased neurite outgrowth without additional stimuli (Figure 3J), while Panx1EGFP overexpression inhibited neurite extension in N2a cells induced to differentiate (Figure 3K).

**Figure 4:**
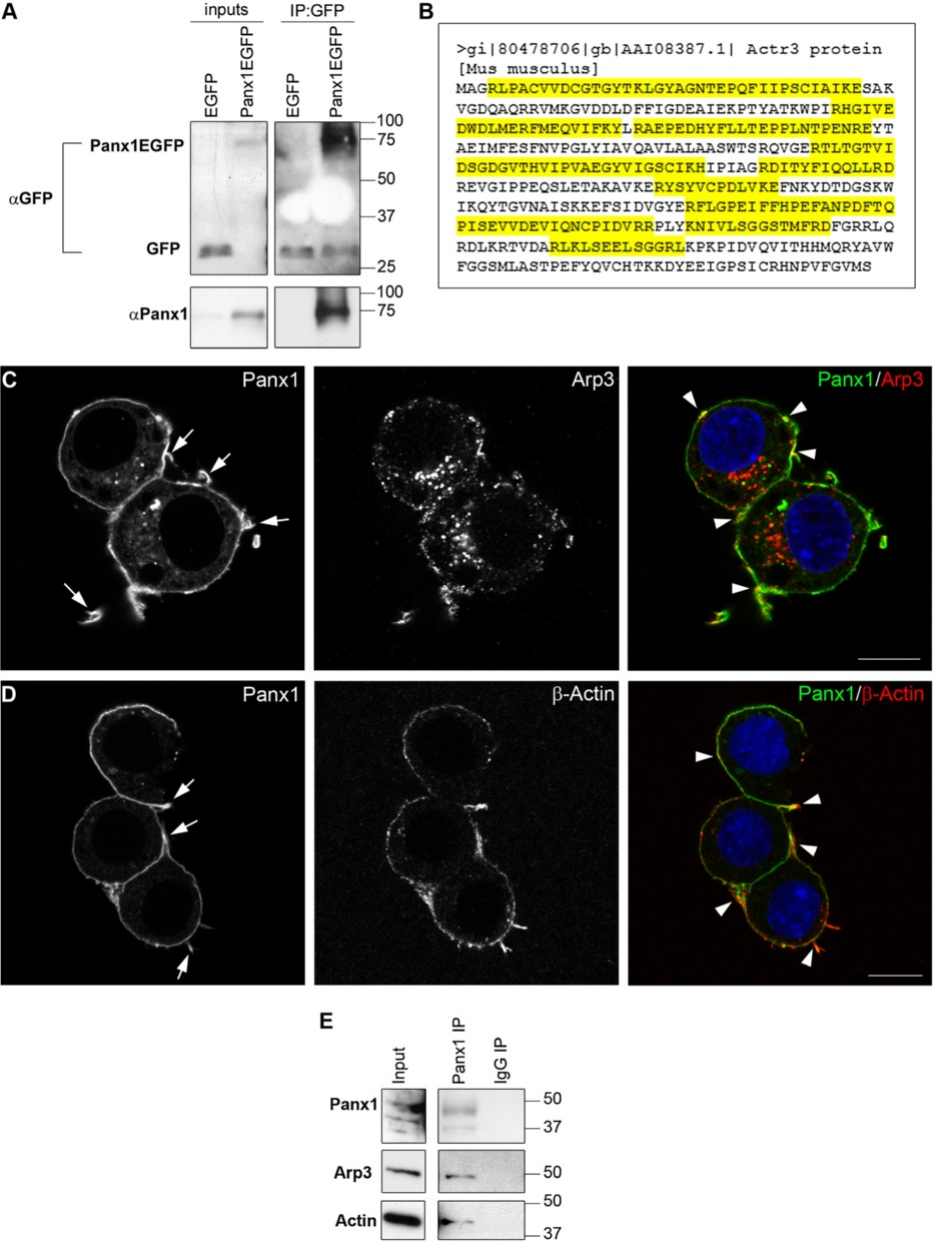
**Panx1 interacts with the cytoskeletal proteins Arp3 and β-Actin. (A)** Western blot of EGFP immunoprecipitations from Panx1EGFP or control EGFP overexpressing N2a cells. Panx1EGFP ran at the expected molecular weight of ~75 kDa (~50 kDa Panx1 + 25 kDa EGFP), and could be visualized with both Panx1 and GFP specific antibodies. Samples were analyzed by LC-MS/MS for Panx1EGFP specific interacting proteins. **(B)** Sequence coverage of the Panx1EGFP interactor, Arp3, from peptides identified in two of three independent replicates of N2a transfection, immunoprecipitation and LC-MS/MS. Peptides identified are highlighted in yellow, and cover 48.8% of the sequence. **(C)** Confocal micrograph of N2a cells overexpressing Panx1EGFP and immunostained for Arp3. Areas of overlap are indicated by arrowheads. Areas of high Panx1EGFP localized to cellular processes are indicated by arrows. **(D)** Confocal micrograph of N2a cells overexpressing Panx1EGFP and immunostained for the β-Actin. Areas of overlap are indicated by arrowheads. Areas of high Panx1EGFP localized to cellular processes are indicated by arrows. **(E)** Western blot of an immunoprecipitation for Panx1 from untransfected N2a cells. The expected Panx1 band is present at ~50 kDa, as well as a lower band that likely corresponds to another glycosylation species. Arp3 and β-Actin were confirmed to interact with endogenous Panx1.

Current knowledge points to close links between Panx1 and cytoskeletal dynamics, and the cell behaviours in which Panx1 appears to be involved in the context of neurogenesis (proliferation, migration, neurite outgrowth) are all tightly linked to the cytoskeleton. To better understand the interface between Panx1 and the cytoskeleton and to determine whether this underlies Panx1 regulation of VZ NSC/NPCs we set out to uncover novel Panx1 interactors using an unbiased proteomics strategy; we are the first group, to our knowledge, to do so. We therefore performed immunoprecipitations from N2a cells overexpressing Panx1-EGFP or EGFP as control (Figure 4A). The EGFP tag does not affect the trafficking or functioning of the Panx1 [6,15,16] and therefore was deemed suitable for use in identification of interactors. The identification of interactors was performed by high-performance liquid chromatography coupled to tandem mass spectrometry (LC-MS/MS). All proteins precipitated by the EGFP tag alone were excluded from further analysis.

We discovered several novel interactions, including several cytoskeleton-related proteins. Gene ontology (GO) analysis (http://www.broadinstitute.org/gsea/msigdb/index.jsp) of hits revealed that 10% of the putative Panx1-interacting proteins amenable to GO analysis could be classified by the generic GO term GO:0005856 aka ‘CYTOSKELETON’ (Additional file 2: Table S1). This GO term refers to ‘*Any of the various filamentous elements that form the internal framework of cells, and typically remain after treatment of the cells with mild detergent to remove membrane constituents and soluble components of the cytoplasm. The term embraces intermediate filaments, microfilaments, microtubules, the microtrabecular lattice, and other structures characterized by a polymeric filamentous nature and long-range order within the cell. The various elements of the cytoskeleton not only serve in the maintenance of cellular shape but also have roles in other cellular functions, including cellular movement, cell division, endocytosis, and movement of organelles.*’

A large number of these newly identified cytoskeleton-associated proteins (14/26) were associated with the GO term collectively known as ‘ACTIN_CYTOSKELETON’ (GO:0015629). Among these, we observed significant overlap of actin-related protein 3 (Arp3; Figure 4B,C) and actin (Figure 4D) with Panx1EGFP by confocal microscopy. Interestingly, as with endogenous Panx1, we also observed high levels of Panx1EGFP localized to neurites and other cellular protrusions resembling filopodia (Figure 4C,D). Finally, endogenous Panx1, actin and Arp3 co-precipitated from N2a cells (Figure 4E). Actin was previously identified as a Panx1 interactor in cells ectopically expressing Panx1 [6]; this confirms that an interaction occurs between the two endogenously expressed proteins.

The novel Panx1 interactor, Arp3, is a major component of the Arp2/3 complex, a seven-subunit protein that plays a major role in the regulation of the actin cytoskeleton (reviewed in Firat-Karalar and Welch, 2011 [17]). A link between Panx1 and actin cytoskeleton rearrangements has previously been described [7]. Arp3 closely resemble the structure of monomeric actin, and one of its functions is to serve as a nucleation site for new actin filaments. Actin and actin-associated proteins, including the Arp2/3 complex, have been shown to be integral in both migration [18,19] and neurite outgrowth [19,20]. Actin polymerization in lamellipodia and filopodia of migrating cells provides the necessary driving force for leading edge protrusion. Furthermore, Arp2/3 complex regulates the actin filaments present in these cellular processes. In fact, Arp2/3 depletion has been shown to significantly reduce filopodia formation in both primary neurons and neuroblastoma cells [21], while alterations to Arp2/3 function cause dysregulation of lamellipodia dynamics [22]. Furthermore, Arp2/3-mediated actin polymerization regulates growth cone mobility and neuritogenesis [23], as loss of Arp2/3 activity causes erratic neurite numbers and extension, as well as increased focal adhesions [21]. Moreover, in agreement with our previously published work illustrating a role for Panx1 in the positive regulation of VZ NSC/NPC proliferation, the actin cytoskeleton has been shown to be indispensable for cell division (reviewed in Firat-Karalar and Welch, 2011 [17]). This includes roles in contractile ring formation, centrosome separation, and spindle positioning. As Panx1 interactors, actin and Arp3 have illustrated a direct connection between Panx1 and the actin cytoskeleton. This further supports the observed role for Panx1 in the actin-associated behaviours of cell migration and neurite outgrowth, as well as that previously published linking Panx1 to cell proliferation.

Altogether, our data expand on our previous findings by demonstrating that Panx1 is expressed in Dcx-positive migrating neuroblasts in adult brain, and is also involved in additional cell behaviours associated with neurogenesis, including migration and neurite outgrowth. Further, our analysis of protein interactions uncovered a novel Panx1 interacting protein, Arp3, a major part of the Arp2/3 complex, which is an important regulator of actin cytoskeletal dynamics in cell proliferation, neuritogenesis and cell migration [17]. We also established that endogenously expressed Panx1 interacts with actin, and discovered that a large proportion of Panx1 interacting proteins are associated with the cytoskeleton. Overall, this study provides novel evidence reinforcing the link between Panx1 and the cytoskeleton, and suggests that this relationship underlies the regulation and function of Panx1 in VZ NSC/NPCs.

## Competing interests

The authors declare that they have no competing interests.

## Authors’ contributions

LAS and LWS devised the study. LWS performed the experiments and data analysis. LAS and LWS wrote and revised the manuscript. Both authors read and approved the final manuscript.

## Supplementary Material

Additional file 1 Detailed description of methods used. Click here for file

Additional file 2: Table S1 GO analysis of Panx1 interactors - gene sets associated with the cytoskeleton. Click here for file
