# Supplementary material for: Panx1 regulates neural stem and progenitor cell behaviours associated with cytoskeletal dynamics and interacts with multiple cytoskeletal elements
Source: Cell Commun Signal. 2013 Aug 21;11:62. doi: 10.1186/1478-811X-11-62 (PMC3765679; doi:10.1186/1478-811X-11-62)
Supplement: Additional file 1 — Detailed description of methods used. [file 1478-811X-11-62-S1.docx]

**SUPPLEMENTAL METHODS**

**Animals and Cell culture**

All procedures were carried out in agreement with the guidelines of the Canadian Council for Animal Care and the University of Victoria Animal Care Committee. Primary NSC/NPC cultures were isolated from postnatal day 0 to 3 (P0 to P3) C57BL/6 mouse periventricular zone and expanded as neurospheres (NSPs) for seven days in vitro (DIV) as described[^1^](#_ENREF_1)^,^[^2^](#_ENREF_2). At DIV7, dissociated NSPs were plated on poly-D-lysine (PDL; 100 μg/mL) coated surface in proliferation media (DMEM/F12, B-27, 2 mM L-glutamine, 100 U/mL penicillin, 100 μg/mL streptomycin, 20 ng/mL human epidermal growth factor, 10 ng/mL fibroblastic growth factor-2; all from Life Technologies, Burlington, Ontario, Canada), or neuronal driving differentiation media (Neurobasal-A, B-27, 0.5 mM glutamax, 100 U/mL penicillin, 100 μg/mL streptomycin; all from Life Technologies, Burlington, Ontario, Canada), and NSPs were collected by washing with DPBS after 5 days. For neurite outgrowth, NSPs were treated with probenecid (1 mM; Panx1 blocker)[^3^](#_ENREF_3) or vehicle 24 hours following dissociation and re-plating, and collected 24 hours later by fixing with 3.7% formaldehyde.

N2a cells were cultured in DMEM/F12 supplemented with 10% fetal bovine serum (FBS), 100 U/mL penicillin, and 100 μg/mL streptomycin (all obtained from Gibco/Life Technologies, Burlington, Ontario, Canada). Where indicated, N2a cells were transfected using jetPEI reagent (Polyplus transfection/VWR; Edmonton, Alberta, Canada) according to the manufacturer’s protocol, with Panx1-EGFP plasmid (a generous gift from Dr. Dale Laird, University of Western Ontario)[^4^](#_ENREF_4)^,^[^5^](#_ENREF_5) or EGFP control plasmid. For knock-down, cells were transfected using Interferin (Polyplus transfection/VWR; Edmonton, Alberta, Canada) with validated Panx1 (targeting 5’-CCACCUUCGAUGUUCUACAUU-3’) or GFP (targeting 5’-AAGCUGACCCUCAAGUUCAUC-3’) siRNAs (Dharmacon/Thermo Fisher Scientific, Lafayette, Colorado, USA) according to the manufacturer’s protocol. For differentiation, N2a cells were plated at 1.3 x 10^4^ cells/cm^2^ on a PDL-coated surface and treated 24 hours later with 10 μM retinoic acid (RA) in low serum media (2% FBS) for 24 hours. Undifferentiated (0 hour) cells were collected as controls. For neurite outgrowth experiments, 1 mM probenecid treatment was performed in normal serum media for 36 hours before collection. Cells were washed 3 times in DPBS and stored at -80°C until lysates were made as described below, or fixed with 3.7% formaldehyde for microscopy.

**GFP Immunoprecipitations and Mass Spectrometry**

Panx1EGFP and EGFP expressing N2a cells were collected 96 hours following transfection for immunoprecipitations. Approximately 4.5x10^7^ cells per condition were homogenized in RIPA buffer (10 mM PBS [150 mM NaCl, 9.1 mM dibasic sodium phosphate, 1.7 mM monobasic sodium phosphate], 1% IGEPAL, 0.5% sodium deoxycholate, 0.1% SDS) supplemented with protease inhibitor cocktail at 1 μL/10^6^ cells (stock: 0.104 mM 4-(2-aminoethyl)benzenesulfonyl fluoride hydrochloride, 0.08 mM aprotinin, 4 mM bestatin hydrochloride, 1.4 mM N-(trans- epoxysuccinyl)-L-leucine 4-guanidinobutylamide, 2 mM leupeptin hemisulfate salt, 1.5 mM pepstatin-A; Sigma-Aldrich) and PMSF at 2 μL/10^6^ cells for 30 minutes, followed by centrifugation for 20 minutes at 12,000 rpm to remove debris. Lysates were pre-cleared for 45-60 minutes with protein-G agarose beads (Roche Applied Science, Mannheim, Germany) at 4°C with shaking, then added to 200 µL protein-G bead suspension cross-linked with 5 µg of αGFP monoclonal antibody (Roche Applied Science, Mannheim, Germany), and incubated overnight at 4°C with shaking. Beads were then washed once with RIPA buffer and twice with PBS, and eluted in 2 bead volumes of 0.5 M ammonium hydroxide/0.5 mM EDTA for 30 minutes at room temperature with shaking. The eluent was dried, and one fifth was rehydrated in RIPA with SDS-PAGE loading dye under reducing conditions (dithiothreitol (DTT) and β-mercaptoethanol) to analyze by Western blotting. Remaining sample was analyzed for potential interactors at the UVIC-Genome BC Proteomics Centre using high performance liquid chromatography coupled to tandem mass spectrometry (LC-MS/MS).

**Endogenous immunoprecipitations**

Approximately 4.5x10^7^ cells per immunoprecipitation were homogenized in TBS (10 mM Tris base, pH 7.4, 150 mM NaCl) with 1% IGEPAL, supplemented with protease inhibitor cocktail at 1 μL/10^6^ cells, PMSF at 2 μL/10^6^ cells and 10 mM sodium orthovanadate for 30 minutes, followed by centrifugation for 20 minutes at 12,000 rpm to remove debris. Supernatants were pre-cleared for 45-60 minutes with protein-A agarose beads (Roche Applied Science, Mannheim, Germany) coupled with ChromPure rabbit IgG (Jackson ImmunoResearch, West Grove, Pennsylvania, USA) at 4°C with shaking, then added to 200 µL protein-A bead suspension cross-linked with 5 µg of αPanx1-CT395 (generously provided by Dr. Dale Laird, University of Western Ontario, Canada) or rabbit IgG, and incubated 1.5 hours at 4°C with shaking. Beads were washed twice with TBS/0.5% IGEPAL and four times with TBS, then eluted in 2 bead volumes of 0.5 M ammonium hydroxide/0.5 mM EDTA for 30 minutes at room temperature with shaking. The eluent was dried and rehydrated in TBS/1% IGEPAL with SDS-PAGE loading dye under reducing conditions (dithiothreitol (DTT) and β-mercaptoethanol) to analyze by Western blotting.

**Western blot analysis**

Western analysis was performed as described[^1^](#_ENREF_1)^,^[^2^](#_ENREF_2). Samples were homogenized in RIPA buffer (10 mM PBS [150 mM NaCl, 9.1 mM dibasic sodium phosphate, 1.7 mM monobasic sodium phosphate], 1% IGEPAL, 0.5% sodium deoxycholate, 0.1% SDS) supplemented with protease inhibitor cocktail at 1 μL/10^6^ cells, PMSF at 2 μL/10^6^ cells, 10 mM sodium orthovanadate and 1 mM EDTA for 30 minutes and centrifuged for 20 minutes at 12,000 rpm to remove debris. For all Western blots, samples were boiled (100°C) for 20 minutes in SDS-PAGE loading dye under reducing conditions (dithiothreitol (DTT) and β-mercaptoethanol).

**Antibodies**

Primary antibodies used were: anti-pannexin 1 C-term (1:200, Invitrogen/Life Technologies, Camarillo, California, USA), anti-pannexin 1-CT395 (1:5000, a generous gift from from Dr. Dale Laird, University of Western Ontario, Canada), anti-β-actin monoclonal (1:160000, Sigma-Aldrich, St Louis, Missouri, USA), anti-Arp3 (1:100, Santa Cruz Biotechnology, Inc, Santa Cruz, California, USA) anti-GFP monoclonal (1:1000, Roche Applied Science, Mannheim, Germany) and anti-DCX (1:4000, Millipore, Billerica, Massachusetts, USA). Secondary antibodies used were horseradish peroxidase (HRP)-conjugated AffiniPure donkey anti-rabbit IgG, HRP-conjugated AffiniPure donkey anti-mouse IgG (both at 1:4000, Jackson ImmunoResearch, West Grove, Pennsylvania, USA), DyLight488-conjugated AffiniPure donkey anti-mouse IgG, DyLight649-conjugated AffiniPure donkey anti-mouse IgG (both 1:600, Jackson ImmuoResearch, West Grove, Pennsylvania, USA) and Alexa Fluor 568-conjugated donkey anti-rabbit IgG (1:600, Invitrogen/Life Technologies, Camarillo, California, USA).

**Microscopy**

P60 mouse brain cryopreservation, serial cryosectioning (20 µm sections) were performed as described[^1^](#_ENREF_1)^,^[^2^](#_ENREF_2)^,^[^6^](#_ENREF_6). Antibodies were diluted in 10 mM PBS supplemented with 0.3% Triton-X-100 and 3% bovine serum albumin. Hoechst 33342 was used as a nuclear counterstain in all imges. Confocal and epi immunofluorescence imaging was performed as previously described[^7^](#_ENREF_7), with a Leica SP8 confocal microscope. For Panx1 siRNA neurite outgrowth experiments, an Incucyte kinetic imaging system was used to capture images (Essen Bioscience, Ann Arbour, Michigan, USA).

**Migration**

Panx1 siRNA knockdown and control N2a cells were grown to confluence before being subject to a scratch wound, and imaged in real time using an Incucyte kinetic imaging system every 2 hours over 80 hours. Incucyte software calculated wound width at each time point for 12 independent biological replicates per condition.

**Neurite Outgrowth**

N2a cell and dissociated NSC/NPC images were analyzed using Adobe Photoshop for cell body length, and number and length of all processes. A process was considered a neurite if it was greater than or equal to the length of the corresponding cell body. Data are based on three independent biological replicates.

**Statistical analyses**

Western blots were quantified using ImageJ 1.45 (http://rsbweb.nih.gov/ij/index.html). Significance was determined using one sample t tests against a hypothetical value of 100% (in percent of control experiments), or unpaired student’s t tests. Variances are reported as standard error of the mean.

**SUPPLEMENTARY REFERENCES**

1. Swayne, L.A., Sorbara, C.D. & Bennett, S.A. Pannexin 2 is expressed by postnatal hippocampal neural progenitors and modulates neuronal commitment. *J Biol Chem* **285**, 24977-24986 (2010).

2. Imbeault, S.*, et al.* The extracellular matrix controls gap junction protein expression and function in postnatal hippocampal neural progenitor cells. *BMC Neurosci* **10**, 13 (2009).

3. Silverman, W., Locovei, S. & Dahl, G. Probenecid, a gout remedy, inhibits pannexin 1 channels. *Am J Physiol Cell Physiol* **295**, C761-767 (2008).

4. Bhalla-Gehi, R., Penuela, S., Churko, J.M., Shao, Q. & Laird, D.W. Pannexin1 and pannexin3 delivery, cell surface dynamics, and cytoskeletal interactions. *J Biol Chem* **285**, 9147-9160 (2010).

5. Penuela, S.*, et al.* Pannexin 1 and pannexin 3 are glycoproteins that exhibit many distinct characteristics from the connexin family of gap junction proteins. *J Cell Sci* **120**, 3772-3783 (2007).

6. Melanson-Drapeau, L.*, et al.* Oligodendrocyte Progenitor Enrichment in the Connexin32 Null-Mutant Mouse. *J. Neurosci.* **23**, 1759-1768 (2003).

7. Wicki-Stordeur, L.E., Dzugalo, A.D., Swansburg, R.M., Suits, J.M. & Swayne, L.A. Pannexin 1 regulates postnatal neural stem and progenitor cell proliferation. *Neural Dev* **7**, 11 (2012).
